# Exploring the Role of Positive Body Image, Depression Symptoms, Disability and Age in Acceptance of Illness in People with Multiple Sclerosis

**DOI:** 10.3390/jcm14030724

**Published:** 2025-01-23

**Authors:** Edyta Matusik, Barbara Lewicka, Kamila Czepczor-Bernat, Katarzyna Maciejowska, Paulina Kowalcze

**Affiliations:** 1 Department of Rehabilitation, Faculty of Health Sciences in Katowice, Medical University of Silesia, 40-055 Katowice, Poland; 2 Department of Therapeutic Rehabilitation, Upper Silesian Medical Centre, 40-635 Katowice, Poland; paukowalcze@gmail.com; 3 Department of Rehabilitation, Doctoral School, Medical University of Silesia, 40-055 Katowice, Poland; barbara.e.lewicka@gmail.com; 4 Department of Pediatrics, Pediatric Obesity and Metabolic Bone Diseases, Faculty of Medical Sciences in Katowice, Medical University of Silesia, 40-055 Katowice, Poland; k.czepczor.bernat@gmail.com; 5 MA-LEK Clinical, 40-571 Katowice, Poland; kata.maciejowska@gmail.com

**Keywords:** multiple sclerosis, illness acceptance, positive body image, disability, depression symptoms, modifiable predictors, nonmodifiable predictors

## Abstract

**Background:** Multiple sclerosis (MS) is a chronic, neurodegenerative disease leading to a progressive disability that can affect not only the physical but also the mental state of patients. The psychological adaptation to the illness plays an important role in the general effectiveness of treatment. The study aimed to determine modifiable and nonmodifiable correlates of acceptance of illness. **Methods:** A total of 139 Caucasian people with MS (pwMS) (106 females) were included. The Acceptance of Illness Scale (AIS), the Body Appreciation Scale (BAS-2) and the Depression, Anxiety and Stress Scale—21 items (DASS-21), together with sociodemographic and clinical data, including the Expanded Disability Status Scale (EDSS), were used. **Results:** Pearson correlation analysis showed that all variables (disability status of pwMS, intensity of depression symptoms and positive body image) are significantly related to acceptance of illness. After including all the predictors in the regression analysis, all the correlations turned out to be significant (except for multiple sclerosis disability rating). Both age and the severity of depressive symptoms decrease the level of acceptance of the disease. An increase in the level of positive body image positively affects the level of acceptance of the disease. **Conclusions:** The acceptance of the disease is correlated with both modifiable and nonmodifiable factors. The work on modifiable factors should be taken into account to create a complex program of rehabilitation and treatment for MS patients.

## 1. Introduction

Multiple sclerosis (MS) is a chronic inflammatory demyelinating disease of the central nervous system (CNS) associated with neurodegenerative processes and inadequate remyelination. Multiple sclerosis is a clinical diagnosis and can only be made in the presence of neurological symptoms resulting from the destruction of oligodendrocytes, myelin sheaths, neurons and axons [1]. Another essential element is a demonstration that the disease process is ‘diffuse in time and space’. The emergence of new symptoms and/or foci of inflammatory/demyelinating activity and the involvement of different parts of the CNS are characteristic of the disease [2]. At the time of diagnosis, the patient becomes forever a ‘person living with multiple sclerosis’ [3]. The disease usually begins between the ages of 20 and 40, when people are at the peak of their careers, and is more common in women [4]. For more than 20 years, there have been significant breakthroughs in the treatment of MS, which have partially altered the so-called natural history of the disease and changed the fate of patients [5]. Nevertheless, MS remains an incurable disease. Even if there are no signs or symptoms at any given time, a young adult will always live with the knowledge of the unpredictable course of the disease and the inevitable increasing disability [6].

In addition to the most common symptoms, such as muscle weakness, sensory loss, impaired motor coordination, chronic fatigue and vision, memory and speech problems, changes in mood and affective state have long been recognized in multiple sclerosis. Depression is the most common mental disorder in MS. The estimated prevalence of major depression in people with MS at any time during the course of the disease is around 15–30 per cent, and 40–60 per cent for lifetime prevalence, which is three times higher than in the general population [7,8,9].

Over time, patients adapt to the disabilities and consequences of the disease with varying degrees of success [10]. For some, living with multiple sclerosis can be like balancing on a razor’s edge. Others cope well, experiencing only transient symptoms for many years [8,11]. The experiences and feelings of people with multiple sclerosis are often more positive than clinicians or others expect [12]. Despite the obvious challenges faced by people with MS, most adapt successfully to the disease, with positive results in self-report measures of the quality of marital and family relationships, relationships with friends, objective measures of emotional functioning, suicidal ideation and self-esteem and direct self-report measures of current coping [13,14].

Such a relationship can be seen in the group of patients who have received professional psychological help at some point since being diagnosed with multiple sclerosis. In contrast, those who are not coping well in different areas of their lives and have difficulty accepting the disease report a need for professional help [15].

It seems that defence mechanisms that influence adaptive behavior play a major role in disease acceptance [10], whether it is mental reconciliation and adaptation to potential limitations, or being ‘here and now’ and trying to fulfil pleasures and enjoy life [16].

Beauty, health and independence are important to young people today. These qualities are not associated with chronic illness. Disability in the course of MS harms body image perceptions. Patients often report dissatisfaction and even shame about their bodies [17]. Importantly, many previous studies have focused on negative body image in patients with MS [18,19], ignoring positive body image (especially in the context of acceptance of illness), which is not just the opposite dimension of negative body image [20,21]. Positive body image is based on appreciating one’s body for its functionality and uniqueness and feeling comfortable and confident in one’s own body/skin. This approach to your own body is supported by maintaining a healthy attitude towards food, exercise and self-care (including self-compassion) [20,21]. Paying attention to this aspect of patients’ mental functioning is extremely important because a positive psychological perception of one’s own body is associated with a number of indicators of well-being such as optimism, positive affect, life satisfaction and self-compassion, as well as proactive coping.

The aim of the study was to examine the relationship between acceptance of illness (explained variable) and its nonmodifiable predictors (i.e., age, disability status in terms of multiple sclerosis) and modifiable predictors (intensity of depression symptoms and positive body image) in patients with multiple sclerosis. It was assumed that significant predictors of acceptance of illness among patients with MS would be age (negative relationship), disability status in terms of multiple sclerosis (negative relationship), intensity of depression symptoms (negative relationship) and positive body image (positive relationship). Referring to the hypothesis and describing the relationships in even more detail—a lower level of intensity of depression symptoms and a higher level of positive body image (both modifiable predictors) would be associated with higher acceptance of illness, after accounting for the effects of nonmodifiable predictors (i.e., age, disability status in terms of multiple sclerosis).

## 2. Methods

### 2.1. Participants and Procedure

We recruited persons with MS by word of mouth and through advertisements in the community, support groups and neurology clinics. The sample size was calculated (effect size = 0.15, power = 0.95, α = 0.05). A power analysis (using the G*Power tool version 3.1.9.7) indicated that the study should involve a minimum of 129 participants. In this study, 139 patients with multiple sclerosis were involved who met the following inclusion criteria: age above 18; residence in Poland; a confirmed diagnosis of relapsing–remitting or secondary multiple sclerosis following the 2017 update to the McDonald criteria; no relapse within the past 30 days; no current alcohol/drug abuse; absence of MS-related cognitive problems; and no medical conditions, such as cardiac diseases, endocrine disorders, musculoskeletal system diseases, current glucocorticoid therapy or respiratory diseases. All patients were on disease-modifying treatment. Recruited patients were on low/moderate-efficacy treatment and high-efficacy treating agents, with the exception of cladribine and alemtuzumab. The number and percentage for each disease-modifying treatment is unknown because these data were not collected and included in the analysis. None of the patients, in response to the question on comorbidities, stated that they had a diagnosis of depression and were therefore on medication or psychotherapy. Each participant wishing to take part in the study received a verbal briefing and a detailed description of the study. Each patient was given sufficient time to decide whether to participate in the study. The patient completed the tests on their own, but always in the presence of a member of the research team, and could receive additional clarification if in doubt. The time required to complete the tests was sufficient to successfully complete the task but not too slow. There were no significant differences between patients in the time allotted to complete the tests.

This study was performed in line with the principles of the Declaration of Helsinki. Approval was granted by the Bioethics Committee of the Silesian Medical University (no. BNW/NWN/0052/KB/123/24 and date of approval: 11 June 2024). Patients were interviewed individually during a scheduled routine clinic visit or at support group meetings.

Patients were ensured of the confidentiality of their data. In describing the results of our study, we used the STROBE checklist for cross-sectional studies.

### 2.2. Measures

#### 2.2.1. The Acceptance of Illness Scale

To measure acceptance of illness, participants were asked to complete the Acceptance of Illness Scale (AIS) [22,23]. It involves 8 items which make up a total score. Our results are interpreted as follows—the higher scores reflected a greater acceptance of illness. All items were assessed on a 5-point scale (from 1—strongly agree to 5—strongly disagree). In our analysis, the Cronbach’s alpha coefficient (internal consistency) was 0.92.

#### 2.2.2. The Depression, Anxiety and Stress Scale (DASS-21)

To measure the intensity of depression symptoms, participants were asked to complete the Depression, Anxiety and Stress Scale (DASS-21) [24]. This scale evaluates one’s emotional state over the previous week and involves 21 items and three subscales: (a) depression, (b) anxiety and (c) stress. The presented analysis focused only on the intensity of depression symptoms, and our results are interpreted as follows—the higher scores reflected a greater level of depression symptoms. It is worth remembering that the DASS is based on a dimensional (intensity evaluation) rather than a categorical conception of psychopathology (disorder vs. no disorder). The scores therefore indicate the degree to which symptoms are experienced (rather than having diagnostic cut points). All items were assessed on a 4-point scale (from 0—did not apply to me at all to 3—applied to me very much or most of the time). In our analysis, the Cronbach’s alpha coefficient (internal consistency) was 0.92.

#### 2.2.3. The Body Appreciation Scale-2

To measure positive body image, participants were asked to complete the Body Appreciation Scale-2 (BAS-2) [20,25]. This scale evaluates body appreciation and only involves a total score. Our results are interpreted as follows—the higher the score, the higher positive body image (body appreciation). All items were assessed on a 5-point scale (from 1—never to 5—always). In our analysis, the Cronbach’s alpha coefficient (internal consistency) was 0.96.

#### 2.2.4. The Expanded Disability Status Scale (EDSS)

This measure was used to assess disability status in terms of multiple sclerosis [26]. It was performed by an experienced and neurostatus-certified neurologist (one of the authors of this article). On this scale, a score reflecting the level of neurological disability is reported (from 0.0—essentially normal to 9.0—restricted to bed). In this context, the scale ranges from EDSS 0 (normal neurologic examination) to 5 (disability severe enough to impair full daily activities) to 10 (death due to MS) in half-point increments from EDSS 1. Due to the nature of the scale, standard reliability coefficients such as Cronbach’s alpha cannot be calculated for it.

#### 2.2.5. Sociodemographic Survey with Questions About Health Status

It was used to collect data such as age, gender, abode, education and marital status. Additionally, patients were interviewed individually and they were asked a number of questions about their health related to MS (details about the time and age of diagnosis, number of relapses and type of MS).

### 2.3. Statistical Analysis

To verify the hypothesis, hierarchical regression was used, and its main assumptions were met. Tolerance was higher than 0.2 and variance inflation factors were not higher than 10 [27,28]. In our regression model, acceptance of illness was a criterion variable (explained variable). As a first step, we entered age. As a second step, we entered disability status in terms of multiple sclerosis, and, as a third step, we entered intensity of depression symptoms and positive body image. Thereby it was possible to assess the extent to which disability status in terms of multiple sclerosis, intensity of depression symptoms and positive body image incrementally predicted acceptance of illness, after accounting for variance associated with the other variables (age).

## 3. Results

### 3.1. Participants

There was a total of 147 patients with a confirmed diagnosis of multiple sclerosis who agreed to participate in the study. Ultimately, 139 patients completed the questionnaire and met the inclusion criteria. The 106 Caucasian women and 33 Caucasian men in the study were residents of the Silesian province. Women were the majority in the study group—76.26%—consistent with the higher prevalence of MS in women, at a ratio of 3:1. Most of them were urban residents (76.23%). Most participants were married (66.91%). Of the participants, 37.41% had university-level education and 31.66% had intermediate-level education. The largest percentage of patients (82.01%) were patients with the relapsing–remitting form of MS. The general distribution of socioeconomic and clinical factors is summarized in Table 1.

### 3.2. Preliminary Analyses

Pearson correlation analysis was performed as the preliminary analysis for the regression model (Table 2). This analysis showed that all variables are significantly related to acceptance of illness.

### 3.3. Regression Analyses

The regression analyses results are shown in Table 3. To summarize our results and referring to the last (third) step in the regression, after taking into account all the predictors, (a) all relationships (except for the one with disability status in terms of multiple sclerosis) turned out to be significant. (b) With increasing age and intensity of depression symptoms the level of acceptance of illness decreases, and as the level of positive body image increases, the level of acceptance of illness increases.

## 4. Discussion

The aim of the study was to assess modifiable and nonmodifiable predictors of acceptance of illness in patients with multiple sclerosis. It was assumed that significant predictors of acceptance of illness among patients with MS would be (a) nonmodifiable predictors, such as age (negative relationship) and disability status in terms of multiple sclerosis (negative relationship), and (b) modifiable predictors, such as the intensity of depression symptoms (negative relationship) and positive body image (positive relationship). Regarding our hypothesis, the regression analysis showed that, ultimately, an increasing level of acceptance of illness can be predicted based on a decrease in the level of age, as well as a decrease in the level of intensity of depression symptoms and an increase in the level of positive body image. These results are therefore partially consistent with our hypothesis and confirm the assumptions regarding age (negative relationship), intensity of depression symptoms (negative relationship) and positive body image (positive relationship), but not those about disability status in terms of multiple sclerosis.

Acceptance of illness in a chronic disease such as multiple sclerosis is an important step for the overall well-being of patients. The psychological adaptation to the illness plays an important role in the general effectiveness of treatment [29]. Our results show the importance of modifiable aspects, which highlights the actual possibility of providing help that would change the patient’s attitude.

The first variable taken into account in the study is the age of patients. Differences between older and younger patients with MS encompass several aspects, including disease progression, symptoms, treatment response and psychosocial impacts. Generally, young patients have a more relapsing–remitting course with periods of remission and fewer immediate severe disabilities. They might experience more acute relapses but often recover better from these episodes [30]. They may struggle with career interruptions and educational impacts and they face unique challenges related to family planning and child-rearing [31]. As indicated, each age group has different challenges and issues to cope with. However, most of the studies show that the quality of life as well as acceptance of illness decrease with age [6,29,32]. Our data show that the average duration of the disease was 11 years and the average age of our patients was 42 years. We can conclude that the older the patient, the longer they will have multiple sclerosis. Longer disease duration is associated with a higher number of exacerbations, changes in central nervous system imaging and an increase in depressive symptoms. These factors have a negative impact on the course of the disease and acceptance of the disease. Also, body image correlates with acceptance of the illness. With age, acceptance of one’s own body is lower and may be associated with acceptance of the illness. In a study by Sabanagic-Hajric et al., both physical and mental health were better in a group of younger patients [33].

Some of the studies emphasize that the neurological state is the most important predictor of acceptance of illness and the general quality of life of MS patients [6]. In our study, the EDSS loses its importance after adding psychological factors to the regression analysis. According to the findings, patients with positive attitudes towards their own body image accept the illness more, even if their neurological status is deteriorating. The claim corresponds with another study performed by researchers in Bosnia. In their research, they estimated the relationship between health-related quality of life and selected demographic and clinical parameters. The results indicated that quality of life is influenced not only by disability itself but rather by interactions of a range of physical, psychological and social factors [34]. The co-occurring impairments in patients with MS that we chose to analyze are depressive symptoms. The chronic nature of the illness, the uncertainty regarding disease progression and the physical disability associated with it contribute to these symptoms. Neuroinflammation and neurodegeneration in MS can also directly impact brain regions involved in mood regulation [35]. The study conducted in Iran indicated that age, economic status and physical activity levels played roles in the levels of depression symptoms experienced by patients [36]. These findings, together with our data, highlight the importance of mental health care in MS centers. Published data from the Polish Multiple Sclerosis Society indicate that 41% of patients report the need for psychological counseling even at the stage of disease diagnosis or treatment decision-making. Patients who have received adequate support from a neurologist or neurology nurse do not report such need [37]. The DASS-21 test used in the publication can be administered and interpreted by non-psychologists. It serves as a useful tool for researchers investigating the nature, etiology and mechanisms of emotional disorders [24]. It is suitable for screening and can support the neurologist in determining whether a patient needs psychological intervention.

Body image issues are a significant concern for patients with MS, influencing their overall mental health and quality of life. In some individuals, a progressive disability can cause changes in their perception of their own bodies [38]. A study by Lo Buono et al. found that in MS patients, body image dissatisfaction is significantly correlated with higher levels of disability, lower self-esteem and higher depression and anxiety. These findings suggest that how patients perceive their bodies can significantly impact their mental health and overall well-being. For instance, there was a positive correlation between body image dissatisfaction and depressive symptoms [18]. In another study, Stevens et al. found that a significant number of patients experience moderate-to-severe concerns about their body image. Factors such as the severity of the disease, type of MS and the duration of the illness were all associated with greater body image issues. The study also emphasized that interventions aimed at improving body image could potentially reduce depressive symptoms and enhance the quality of life for these patients [19]. This finding corresponds indirectly with our finding of a great correlation between body image and acceptance of illness.

Between many strategies indirectly improving the acceptance of illness, in the Netherlands, a direct approach was used. A small study was performed on six female patients. The patients underwent Acceptance and Commitment Therapy, which involved eight weekly sessions of two hours, led by a certified psychologist or Acceptance and Commitment Therapy therapist. After completing the therapy, five participants showed statistically significant increases in quality of life, and acceptance increased in two patients [39]. Despite the limitations of the study (the number of participants), it could be an interesting line of further research.

Multiple sclerosis (as a chronic illness) may have a significant impact on an individual’s body image which can be related to the physical, emotional and psychological effects of this illness [18,19,40,41]. It should be noted that MS can often lead to changes in physical appearance, mobility and the ability to perform daily activities and it can affect self-perception and body image [18,19,40,41]. Adaptive strategies supporting acceptance of the disease may therefore include [40,41,42,43,44] (a) understanding the nature of MS and its symptoms (which can support individuals to more effectively manage their condition and reduce depression and anxiety symptoms), (b) concentrating on what their body can do rather than what it cannot (including acceptance of the body’s limitations resulting from the disease based on an attitude of self-compassion, e.g., techniques from Acceptance and Commitment Therapy), (c) taking care of other important needs of the body (i.e., physical activity or nutrition), approaching the body with respect, accepting its needs and approaching it with mindfulness (including openness to the use of adaptive devices that can help with movement and maintaining independence in engaging in activities that bring pleasure—a technique from Acceptance and Commitment Therapy), (d) taking care of rest and relaxation (e.g., using meditation and relaxation techniques to help maintain a positive relationship with the body, which may be an important factor in protection against the development of depression), (e) working with negative thoughts (e.g., using Cognitive Behavioral Therapy techniques), which are important factors increasing the risk of depression and deterioration of body image, and (f) building a support network.

## 5. Conclusions

Acceptance of illness can be explained by both modifiable and nonmodifiable factors associated with MS. The psychological aspects can strongly affect the overall acceptance of illness. As modifiable predictors, they can and should be treated pharmacologically or psychologically. Holistic approaches in MS treatment are needed.

## 6. Limitations

Importantly, when discussing the limitations of this study, it is important to mention the following: (a) this is a cross-sectional study (not a longitudinal study or an intervention/experiment with a control group), so we can draw limited conclusions about the relationships between variables; (b) the measurement of many variables was performed using self-report tools (replicable methods such as answered questionnaires); (c) we did not consider aspects such as professional activity or ways of spending free time (hobbies, sports); (d) due to sample size limitations, the analysis did not take into account cut-off points for each factor of DASS-21; (e) due to sample size limitations, the analysis did not include factors such as MS types, time since diagnosis, social status, age or medication; (f) disease-modifying therapy was not included in the analysis; and (g) some other important variables were not collected and/or not included in the analysis (i.e., relapse-related characteristics, e.g., time between relapses, level of anxiety/stress). However, further research is needed to confirm the significance of the listed variables and their interactions.

## Figures and Tables

**Table 1:** Sociodemographic and clinical characteristic data of the participants.

Sociodemographic Characteristics		
Age at the Time of the Study (M Mean Years (SD) 41.83 (10.65))		
N (%)		
Gender	Women	106 (76.26)
	Men	33 (23.74)
Abode	City	106 (76.26)
	Village	33 (23.74)
Education	Vocational	14 (10.07)
	Secondary	44 (31.66)
	Higher	52 (37.41)
	Other	29 (20.86)
Marital Status	Single	25 (17.99)
	Marriage	93 (66.91)
	Divorce/separation	8 (5.76)
	Informal relationship	10 (7.19)
	Widow/widower	1 (0.72)
	Other	2 (1.44)
		Clinical Characteristics
		M (SD)
Number of relapses since diagnosis		3.94 (4.29)
Age at diagnosis of multiple sclerosis		31.00 (9.48)
Time since diagnosis in years		10.84 (8.53)
Disability status EDSS		2 (1.63)
		N (%)
Types of MS ^1^	Relapsing–remitting MS (RRMS)	114 (82.01)
	Primary progressive MS (PPMS)	11 (7.91)
	Secondary progressive MS (SPMS)	13 (9.35)

^1^ One patient did not answer this question. Abbreviation: EDSS—Expanded Disability Status Scale.

**Table 2:** Correlation analysis (Pearson’s r).

	Age	Disability Status in Multiple Sclerosis	Intensity of Depression Symptoms	Positive Body Image
Acceptance of illness	*r* = −0.30 ***	*r* = −0.36 ***	*r* = −0.39 ***	*r* = 0.43 ***

Note. *** *p* < 0.001.

**Table 3:** Results of hierarchical regression analysis for the prediction of acceptance of illness.

		Acceptance of Illness				
Step	Variables	B	SE	*β*	*T*	*p*
1		*F*(1, 138) = 13.53, *p* < 0.001, Adj. *R*^2^ = 0.08				
	Age	−0.27	0.07	−0.30	−3.68	<0.001
2		*F*(2, 138) = 11.75, *p* < 0.001, Adj. *R*^2^ = 0.14, Δ*Fp* < 0.01				
	Age	−0.13	0.08	−0.15	−1.55	>0.05
	Disability status in multiple sclerosis	−1.67	0.55	−0.29	−3.03	<0.01
3		*F*(4, 138) = 15.64, *p* < 0.001, Adj. *R*^2^ = 0.30, Δ*Fp* < 0.001				
	Age	−0.20	0.08	−0.22	−2.55	<0.05
	Disability status in multiple sclerosis	−0.87	0.52	−0.15	−1.68	>0.05 ^1^
	Intensity of depression symptoms	−0.20	0.08	−0.23	−2.64	<0.01
	Positive body image	2.48	0.89	0.25	2.80	<0.01

Note. ^1^ *p* = 0.096.

## Data Availability

The data presented in this study are available on request from the corresponding author.
